# Vegetation Length is Associated with Long-term Survival in Patients Treated Surgically for Infective Endocarditis

**DOI:** 10.31083/j.rcm2510354

**Published:** 2024-09-30

**Authors:** Jing-bin Huang, Sheng-jing Liang, Chang-chao Lu, Zhao-ke Wen

**Affiliations:** ^1^Department of Cardiothoracic Surgery, The People’s Hospital of Guangxi Zhuang Autonomous Region, and Guangxi Academy of Medical Sciences, 530021 Nanning, Guangxi, China

**Keywords:** endocarditis, surgery, greater than 10 mm, vegetation length

## Abstract

**Background:**

The impact of vegetation length on therapeutic decision-making and prediction of long-term survival of patients with infective endocarditis is a highly topical issue. The aim of the study was to clarify the impact of vegetation length greater than 10 mm on long-term survival treated surgically for infective endocarditis.

**Methods:**

Patients treated surgically for infective endocarditis in our hospital from January 2006 to November 2022 and were successfully followed up were included in the retrospective analysis.

**Results:**

814 survivors discharged from our medical center were successfully followed up to the date of death or the end date of the research and allocated to a group with vegetation length <10 mm (n = 432) or ≥10 mm (n = 382). The average follow-up time was 75.1 ± 1.8 months. Multivariate analysis indicated vegetation length ≥10 mm was associated with 1-year and 5-year mortality. Multivariate analysis of Cox regression identified vegetation length ≥10 mm to be associated with all-time mortality. Multivariate analysis identified male gender, long time between symptoms and surgery, more preoperative left ventricular ejection fraction (LVEF) and more preoperative aortic regurgitation to be associated with vegetation length ≥10 mm in infective endocarditis.

**Conclusions:**

Our study indicated that vegetation length ≥10 mm was associated with long-term survival in patients treated surgically for infective endocarditis.

## 1. Introduction

Infectious endocarditis (IE) is a complicated intravascular infection related to endothelial injury, with potential complications threatening life, with an annual mortality rate of up to 40%. If left untreated, IE is almost always fatal [1, 2, 3, 4, 5].

An increasing number of elderly people have suffered from degenerative heart valve disease in the past 30 years, with an increase in *Staphylococcus* infection, leading to an increase in the incidence of IE [6]. IE patients usually have severe conditions due to involvement in various organ systems and impaired hemodynamics. Even in experienced centers, IE surgery still has the highest mortality rate among all valve diseases [6, 7]. Despite advances in rapid diagnosis, better antimicrobial therapy, management of intensive care, and surgical techniques over the years, surgery remains challenging with a high incidence of complications [8, 9]. Multi center research has reported in-hospital mortality rates of 15–20%, with 1-year mortality rates approaching 40% [6, 7, 10].

Vegetation is a hallmark of IE. It is widely accepted that vegetation length ≥10 mm is the most powerful independent predictor of new embolic events [11, 12, 13]. Investigation of vegetation length has clinical implications for the management of IE. It is generally accepted that vegetation length greater than 10 mm is the strongest independent risk factor of new embolic events. The investigation of vegetation length poses clinical significance for the treatment of infective endocarditis. The impact of the vegetation lengths on therapeutic decision-making and prediction of long-term survival of patients with infective endocarditis is a highly topical issue. The aim of the study was to clarify the impact of vegetation length greater than 10 mm on long-term survival when treated surgically for infective endocarditis. We hypothesize that vegetation length greater than 10 mm predicts worse long-term survival when treated surgically for infective endocarditis [4, 5, 6, 7, 8].

## 2. Study Population and Methods 

### 2.1 Design

We retrospectively studied patients treated surgically for infective endocarditis from January 2006 to November 2022 at our medical center by reviewing medical records.

### 2.2 Diagnosis

Patients were diagnosed based on the modified Duke criteria [14]. We reviewed surgical and pathological results to affirm diagnosis preoperatively.

### 2.3 Criteria of Eligibility 

**Fig. 1.**
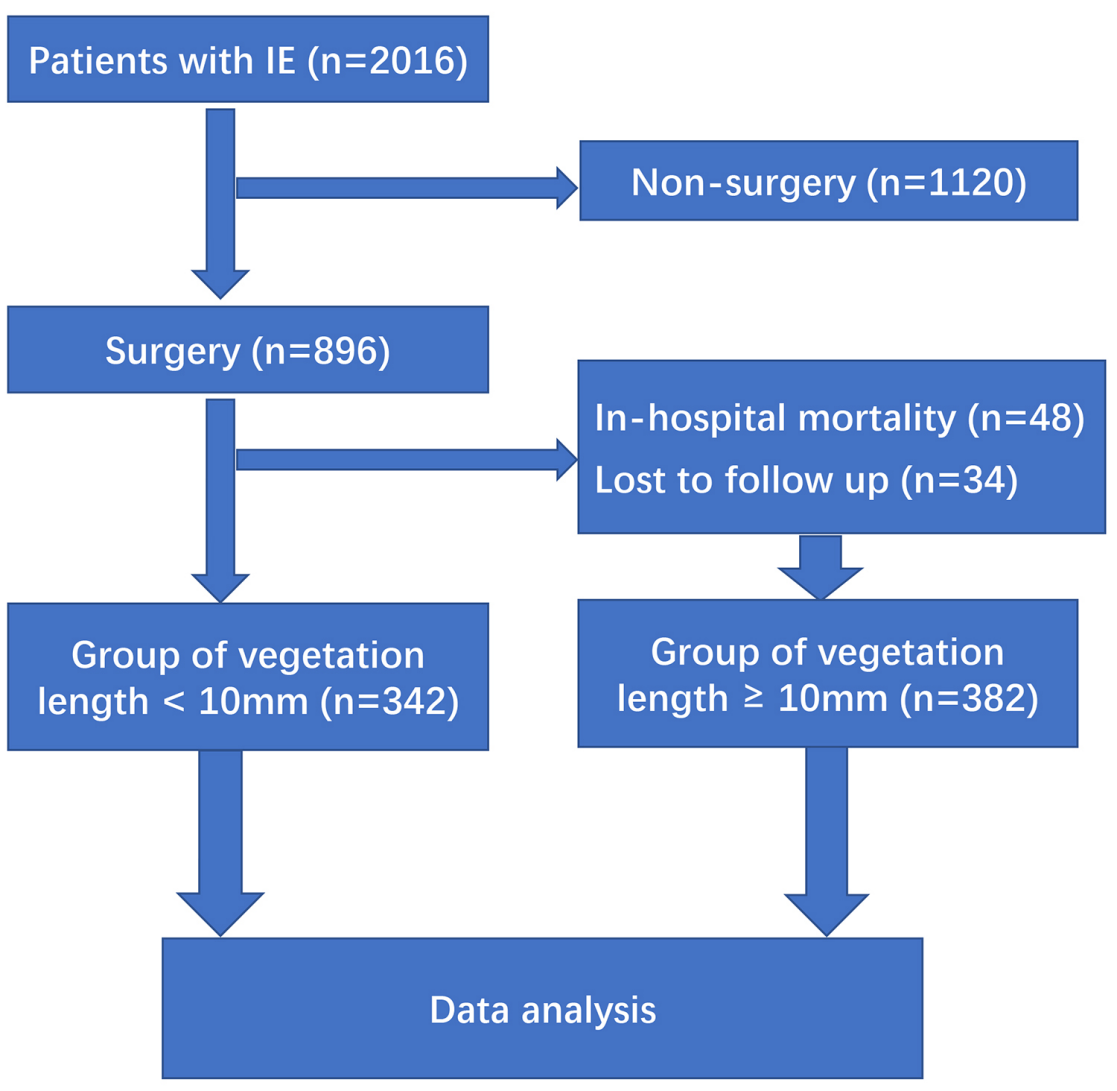
**Flow chart of patients**. IE, infectious endocarditis.

The inclusion criteria consisted of patients treated surgically for IE from January 2006 to November 2022 at our medical center discharged and followed up successfully to the date of death or the end of the research. Exclusion criteria included in-hospital death and the patients being lost to follow up (Fig. 1).

### 2.4 Variables Analyzed 

#### 2.4.1 Variables in Supplementary Data were Analyzed

Time between symptoms and surgery refers to the time from the onset of symptoms to the surgery date.

In-hospital mortality refers to death within 30 days after surgery or during the same hospitalization period.

#### 2.4.2 Follow-up

We monitored all discharged survivors until the end of the study. All patients were scheduled to undergo echocardiography, electrocardiogram, and chest X-ray examinations every 3 to 12 months at the outpatient department. In the final follow-up, we contacted the patients via phone or WeChat, or conducted interviews directly at the outpatient department. Survivors discharged from our medical center and successfully followed up to the date of death or the end date of the study were allocated to either the group with vegetation length <10 mm or ≥10 mm [4, 5, 6, 7, 8].

### 2.5 Statistical Analyses

Continuous variables are presented as mean ± standard error (SE). We performed normality tests on all variables using the Kolmogorov Smirnov test and used Kaplan-Meier method to assess survival rate in the study. The chi square test, Wilcoxon rank sum test, or Kruskal Walls test (depending on the situation) are used to evaluate the relationship between variables preoperative and selected variables intraoperative and postoperative. We used a contingency table method and logistic regression analysis to evaluate the relationship with perioperative risk factors. The survival rate was analyzed using analysis of Kaplan-Meier, and the survival rates differences between groups were tested using a logarithmic rank test. We also used a multivariate Cox proportional risk model. A *p*-value less than 0.05 was deemed statistically significant. Analyses were completed all using IBM SPSS 24.0 software (IBM SPSS Inc., Armonk, NY, USA).

## 3. Outcomes

896 patients were treated surgically for IE during the study period. 48 operative deaths occurred (48/896, 5.36%). Non-surgery mortality was 68.57% (768/1120). 34 patients were lost to follow up. 814 survivors discharged from our medical center were successfully followed up to the date of death or the end date of the study and allocated to group with vegetation length <10 mm (n = 432) or ≥10 mm (n = 382) (Tables 1,2).

**Table 1.** **Preoperative, surgical, and follow-up data**.

Variable		Group with vegetation length <10 mm (n = 432)	Group with vegetation length ≥10 mm (n = 382)	*p* value
Preoperative data				
	Male gender, n (%)	293 (61.4%)	247 (73.3%)	<0.001
	Age	38.23 ± 0.71	38.54 ± 0.74	0.765
	Weight, kg	56.55 ± 0.56	55.31 ± 0.69	0.146
	Time between symptoms and surgery, months	2.19 ± 0.12	3.09 ± 0.13	<0.001
	Preoperative LVEDD, mm	61.39 ± 0.50	60.46 ± 0.43	0.163
	Preoperative LVEF, %	59.92 ± 0.37	61.71 ± 0.44	0.002
	Preoperative aortic regurgitation, cm^2^	4.04 ± 0.26	7.12 ± 0.39	<0.001
	Preoperative mitral regurgitation, cm^2^	6.89 ± 0.33	7.62 ± 0.27	0.087
	Preoperative tricuspid regurgitation, cm^2^	4.84 ± 0.27	2.59 ± 0.19	0.465
	Neurological complications before surgery, n	46 (9.6%)	30 (8.9%)	0.720
	Serum creatinine before surgery, µmol/L	75.57 ± 0.97	78.41 ± 1.32	0.078
Operative data				
	Acute kidney injury, n	92 (13.9%)	120 (35.6%)	<0.001
	Aortic occlusion time, minutes	81.18 ± 1.49	91.02 ± 1.82	<0.001
	CPB time, minutes	129.92 ± 2.05	146.45 ± 2.52	<0.001
	Mechanical ventilation time, hours	40.46 ± 2.0	34.15 ± 1.98	0.026
	Length of ICU stay, days	4.91 ± 0.14	4.07 ± 0.13	<0.001
	Postoperative hospital stay, days	18.43 ± 0.25	19.98 ± 0.46	0.002
	Creatinine of serum 24 h after surgery, µmol/L	86.44 ± 1.59	86.92 ± 2.12	0.854
	Creatinine of serum 48 h after surgery, µmol/L	89.26 ± 2.28	102.95 ± 3.08	<0.001
	Balance of fluid on the day of operation, mL	–473.89 ± 36.83	–828.35 ± 38.27	<0.001
	Balance of fluid on 1st day postoperative, mL	–662.5 ± 61.73	–612.7 ± 33.36	0.494
	Balance of fluid on 2nd day postoperative, mL	–582.41 ± 37.28	–653.91 ± 33.46	0.665
	Chest drainage, mL	631.37 ± 19.41	605.81 ± 19.25	0.352
	Postoperative LVEDD, mm	47.34 ± 0.33	47.62 ± 0.38	0.582
	Postoperative LVEF, %	57.74 ± 0.34	59.29 ± 0.42	0.004
	Frozen plasma, mL	617.22 ± 24.19	613.74 ± 21.90	0.916
	Packed red cells, units	2.48 ± 0.12	2.28 ± 0.12	0.235
Follow-up data				
	Length of follow-up, months	77.28 ± 3.0	63.64 ± 2.3	<0.001
	All-time mortality, n	27 (6.3%)	73 (19.1%)	<0.001

LVEDD, left ventricle end-diastolic diameter; LVEF, left ventricular ejection fraction; ICU, intensive care unit; CPB, cardiopulmonary bypass.

**Table 2.** **Operation and causes of in-hospital mortality and complications in infective endocarditis (n = 896)**.

Variable		Value	Mortality
Surgical operation			
In-hospital mortality			5.36 (48/896)
	AVR isolated, %	19.64% (176/896)	1.34% (12/896)
	MVR isolated, %	41.07% (368/896)	1.79% (16/896)
	Double valve operation, %	28.57% (256/896)	2.23% (20/896)
	Bentall + MVR, %	1.79% (16/896)	0
	Tricuspid annuloplasty isolated, %	8.92% (80/896)	0
	ECMO, %	0.33% (3/896)	
Causes of in-hospital mortality, %			
	Paravalvular leak + septicemia + AKI + hepatic failure + cardiogenic shock	3.57% (32/896)	
	Encephalorrhagia	1.79% (16/896)	
Complications			
	Acute kidney injury, %	28.68% (257/896)	
	Mechanical ventilation time >72 h	21.43% (192/896)	
	Liver failure, %	4.35% (39/896)	
	Respiratory failure, %	15.07% (135/896)	
	Ventricular fibrillation, %	3.68% (33/896)	

AKI, acute kidney injury; AVR, aortic valve replacement; MVR, mitral valve replacement; ECMO, extracorporeal membrane oxygenation.

### 3.1 Preoperative Data

Compared with the group with vegetation length <10 mm, the time between symptoms and surgery (3.09 ± 0.13 vs 2.19 ± 0.12 months, *p* < 0.001), preoperative left ventricular ejection fraction (LVEF) (61.71 ± 0.44 vs 59.92 ± 0.37%, *p* = 0.002), and preoperative aortic valve regurgitation (7.12 ± 0.39 vs 4.04 ± 0.26 cm^2^, *p* < 0.001) significantly increased in the group with vegetation length ≥10 mm (Table 1).

### 3.2 Operative Data

Compared with the group with vegetation length <10 mm, aortic occlusion time (91.02 ± 1.82 versus 81.18 ± 1.49 minutes, *p* < 0.001), cardiopulmonary bypass (CPB) time (146.45 ± 2.52 versus 129.92 ± 2.05, *p* < 0.001), postoperative hospital stay (19.98 ± 0.46 versus 18.43 ± 0.25 days , *p* = 0.002), serum creatinine 48h post-surgery (102.95 ± 3.08 versus 89.26 ± 2.28 µmol/L, *p* < 0.001), postoperative LVEF (59.29 ± 0.42 versus 57.74 ± 0.34%, *p* < 0.001) significantly increased in the group with vegetation length ≥10 mm (Table 1).

Multivariate analysis showed that factors are related to vegetation length ≥10 mm in infective endocarditis, including male gender (odd ratio, OR: 1.652, 95% CI: 1.219–2.238, *p* < 0.001), time between symptoms and surgery (OR: 1.169, 95% CI: 1.103–1.239, *p* < 0.001), preoperative LVEF (OR: 12.052, 95% CI: 1.924–36.215, *p* = 0.008), and preoperative aortic regurgitation (OR: 1.165, 95% CI: 1.127–3.257, *p* < 0.001) (Table 3).

**Table 3.** **Analysis of risk factors for infective endocarditis with vegetation length ≥10 mm**.

Model		OR	95% CI	*p* value
Univariate analysis				
	Male gender	1.756	1.310–2.355	<0.001
	Time between symptoms and surgery	1.152	1.086–1.222	<0.001
	Preoperative LVEF	15.360	2.743–86.00	<0.001
	Preoperative aortic insufficiency	1.075	1.051–1.100	<0.001
Multivariate analysis				
	Male gender	1.652	1.219–2.238	<0.001
	Time between symptoms and surgery	1.169	1.103–1.239	<0.001
	Preoperative LVEF	12.052	1.924–36.215	0.008
	Preoperative aortic insufficiency	1.165	1.127–3.257	<0.001

OR, odd ratio; LVEF, left ventricular ejection fraction.

### 3.3 Follow-up Data

The average follow-up time was 75.14 ± 1.80 months (range, 1 to 204). 87 cases (87/814, 10.7%) died within 12 months of discharge due to IE recurrence and cerebral hemorrhage. The latest follow-up data shows that 681 survivors belong to New York Heart Association (NYHA) class I (681/727, 85.0%), and 109 survivors belong to class II (109/727, 15.0%). Length of follow-up (63.64 ± 2.3 versus 77.28 ± 3.0 months, *p* < 0.001) in the group with vegetation length ≥10 mm was statistically significantly less than that in the group with vegetation length <10 mm. Compared with the group with vegetation length <10 mm, all-time mortality (19.1% versus 6.3%, *p* < 0.001) significantly increased in the group with vegetation length ≥10 mm (Table 1).

Multivariate analysis showed that vegetation length ≥10 mm had statistical significance with 1-year (OR: 1.60, 95% CI: 1.107–1.216, *p* < 0.001) and 5-year (OR: 1.193, 95% CI: 1.149–22139, *p* < 001) mortality rates (Table 4).

**Table 4.** **Analysis of the implication of vegetation length on the long-term mortality of infective endocarditis (n = 814)**.

Model		OR	95% CI	*p* value
Univariate analysis of risk factors of 1-year mortality after cardiac operation (n = 87)				
	Vegetation length ≥10 mm	1.173	1.118–1.231	<0.001
Multivariate analysis of risk factors of 1-year mortality after cardiac operation (n = 87)				
	Vegetation length ≥10 mm	1.160	1.107–1.216	<0.001
Univariate analysis of risk factors of 5-year mortality after cardiac operation (n = 100)				
	Vegetation length ≥10 mm	1.124	1.079–1.171	<0.001
Multivariate analysis of risk factors of 5-year mortality after cardiac operation (n = 100)				
	Vegetation length ≥10 mm	1.193	1.149–2.139	<0.001

OR, odd ratio.

**Fig. 2.**
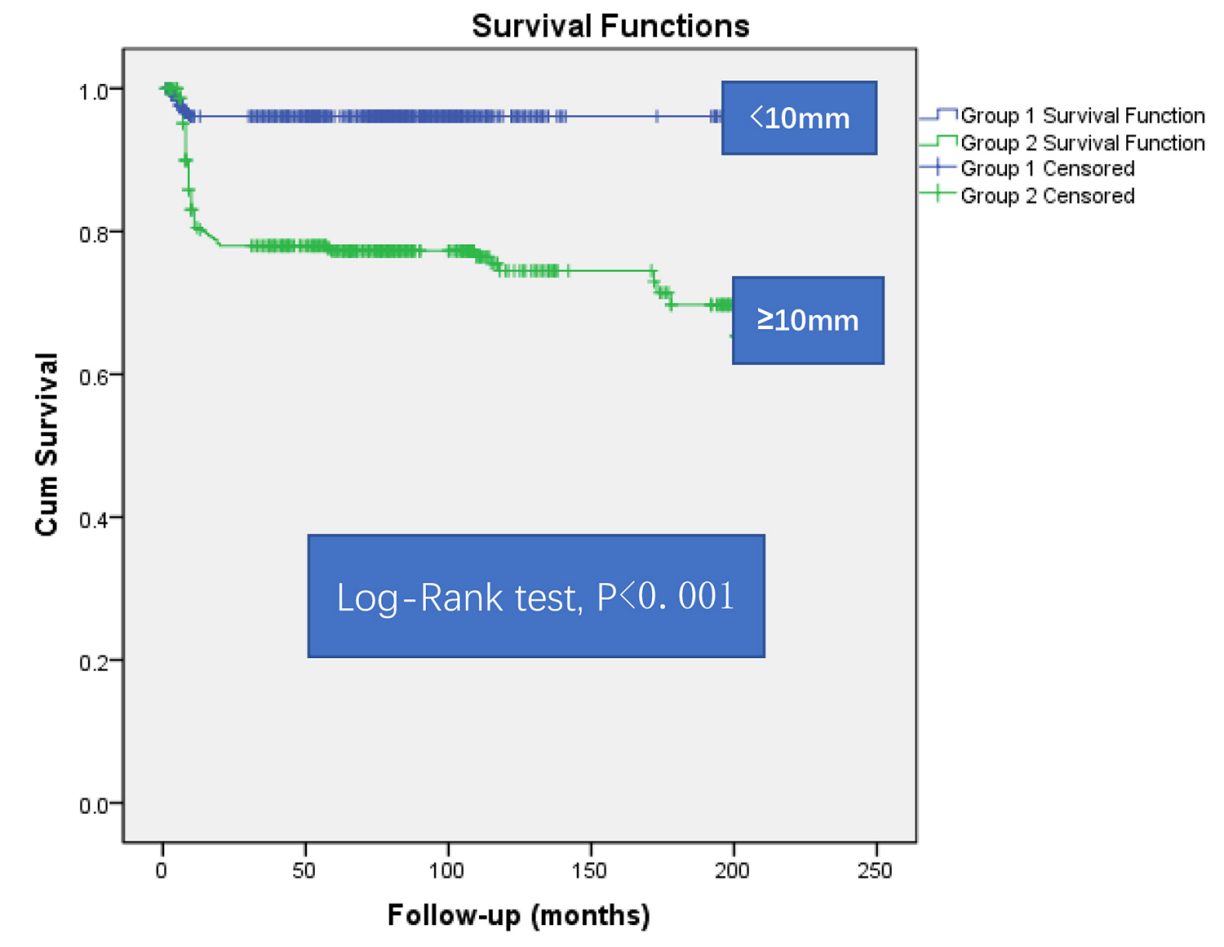
**Kaplan-Meier curve for survival**. Blue line, Group of vegetation length <10 mm; Green line, Group of vegetation length ≥10 mm. Cum Survival, cumulative survival.

The presence of vegetation length ≥10 mm in IE significantly enhances in-hospital mortality and is also a significant risk factor of long-term survival (Log-Rank test, *p* < 0.001) (Fig. 2 and Table 5).

**Table 5.** **Patients at risk**.

Months	50	100	150	200	250
Vegetation length <10 mm	324	102	76	24	0
Vegetation length ≥10 mm	243	90	30	12	0

Multivariate analysis of Cox proportional hazard regression for all-time mortality identified vegetation length ≥10 mm (HR: 2.744, 95% CI: 1.860–4.046, *p* < 0.001), time between symptoms and surgery ≥1 month (HR: 14.061, 95% CI: 4.472–44.214, *p* < 0.001) and cardiopulmonary bypass time ≥120 minutes (HR: 3.766, 95% CI: 2.352–6.032, *p* < 0.001) to be associated with all-time mortality (Table 6).

**Table 6.** **Cox proportional risk regression of all-time mortality (n = 814)**.

Model		HR	95% CI	*p* value
Univariate analysis				
	Vegetation length ≥10 mm	3.264	2.223–4.794	<0.001
	Time between symptoms and surgery ≥1 month	13.341	4.264–41.922	<0.001
	Cardiopulmonary bypass time ≥120 minutes	4.697	2.943–7.497	<0.001
Multivariate analysis				
	Vegetation length ≥10 mm	2.744	1.860–4.046	<0.001
	Time between symptoms and surgery ≥1 month	14.061	4.472–44.214	<0.001
	Cardiopulmonary bypass time ≥120 minutes	3.766	2.352–6.032	<0.001

HR, hazard ratio.

## 4. Discussion

Despite recent advances in diagnosis and treatment, rates of in-hospital mortality of IE still exceeds 20%, and the mortality rate at 3 years still exceeds 30%. Large vegetations predict patients with poor outcomes in left-sided IE [1, 2, 3, 15, 16].

We identified being male, increased time between symptoms and surgery, presence of preoperative LVEF and preoperative aortic insufficiency to all be associated with vegetation length ≥10 mm in infective endocarditis. We found vegetation length ≥10 mm to be associated with increased 1-year and 5-year mortality. Multivariate analysis of Cox regression identified vegetation length ≥10 mm to be associated with all-time mortality.

The valve junction area is the central area of the valve surface most commonly affected. Endocardial injury attracts fibrin and platelet deposits to the injury site, where bacteria settle, resulting in the production of infected vegetation. Therefore, the presence of vegetation is a major criterion for diagnosing infectious endocarditis at Duke University. Vegetation size has been considered a predictive factor for thrombotic mortality and complications. The current guidelines of American Heart Association emphasize that vegetation length larger than 10 mm is a critical value for intervention of surgery and suggest follow-up transthoracic echocardiography following completion of antimicrobial therapy. Given the significant burden of mortality caused by complications of embolization, a more detailed study is needed on the factors that greatly affect the embolization process, such as vegetation size [17, 18, 19, 20, 21].

Several studies have shown that the risk of embolic events is significantly reduced in the second week post starting targeted antibiotic therapy. Timely initiation of appropriate antibiotic treatment is effective in reducing embolic events. After appropriate antibiotic treatment, the vegetation area significantly decreased. However, completely resolved situations are not common. Statistically significant relationship exists between vegetation size, intravenous drug use, and *Staphylococcus* species. Intravenous medication and Staphylococcal endocarditis affect vegetation size and thrombotic complications. Published literature indicates that after at least six weeks of antibacterial treatment, vegetation size often decreases; however, in most cases, persistent residual vegetation usually exists. Based on studies published, residual vegetation does not add to the risk of embolism, death, or recurrence unless their length is >10 mm or their size increases after treatment [22, 23, 24, 25].

In the study conducted by Huang *et al*. [5], vegetation length was significantly correlated with destructive valve annulus, preoperative stroke, acute kidney injury, prolonged mechanical ventilation time (mechanical ventilation time >24 hours), prolonged intensive care unit (ICU) stay (>3 days), in-hospital mortality, and 1-year mortality rate, respectively.

### 4.1 Risk Factors Analysis of Vegetation Length ≥10 mm

The presence of vegetation ≥10 mm is the main factor resulting in embolism, valve regurgitation, and heart failure. Understanding the pathogenesis of vegetation formation contributes to describing the impact of the *Staphylococcus* species on vegetation length. Vegetation is mainly composed of fibrin and platelets, depositing on the endothelium of the heart after some form of damage. It has been shown that *Streptococcus* and *Staphylococcus* strains increase aggregation of platelets more than other pathogens, helping to accelerate vegetation growth. The bacterial density in these vegetation centers is high and their metabolism is inert, reducing the antibacterial effect and leading to the sustained existence of large vegetation [26, 27, 28].

In the present study, we identified the male gender, time between symptoms and surgery, preoperative LVEF and preoperative aortic insufficiency to be associated with vegetation length ≥10 mm in infective endocarditis, which have been reported. The male gender is prone to suffer from infective endocarditis and increased vegetation lengths are more likely, although the reasons have not been fully clarified. The longer the time between symptoms and surgery, the longer the length of vegetation. Therefore, early diagnosis and surgical intervention have significant implications for reducing the time until surgical intervention. Completing a rapid and accurate diagnosis in a short time frame from symptoms to surgery in cases of infective endocarditis remains a central challenge of the disease. The large volume of retrograde diastolic flow with high shear force and turbulence formed by reflux beams, and special types of reflux beams (impact, acceleration, and split) can damage the endocardium of left ventricle, which contribute to the growth of vegetation.

### 4.2 Implications for Surgical Intervention 

The American Heart Association guidelines recommend surgical treatment for patients with severe valve regurgitation and vegetation >10 mm to prevent embolic events [6]. When the vegetation size is larger than 10 mm, especially when it involves the anterior leaflet of the mitral valve, and when it is related to other relevant indications for surgery, surgery can also be considered. The guidelines of the European Society of Cardiology recommend that despite appropriate antibiotic treatment, surgery should be performed on left persistent vegetation >10 mm after one or more embolic events. Surgery can also be considered in isolated left large (>15 mm) vegetation, when there are no other surgical indications [4]. In the absence of complications of uncontrolled infection or heart failure, the use of vegetation length as the sole indication for surgical intervention aimed at preventing embolism is still controversial. Research has indicated that the inter observer variability in estimating vegetation length is too high to guide surgical decisions. In addition to the maximum diameter, there are several other morphological vegetation features, such as attachment width, mobility, shape, and echo density on the surface of the endocardium, which can affect the incidence of embolism. In addition, vegetation location (mitral valve location is more prone to embolism than aortic location) and specific pathogenic microorganisms have been found to be associated with the onset of emboli [17].

The prevention of heart failure, uncontrolled infections, and embolic events is an indication for early IE surgery. The length of vegetation may be one of the reasons for surgery, but it is rarely the only reason. If there was no previous embolism, surgical indications indicate vegetation length >10 mm, as well as some other predictive factors for complex IE (heart failure, uncontrolled infection). Early surgery can prevent stroke in left-sided infective endocarditis. Guidelines of American Association for Thoracic Surgery recommend urgent or even emergency surgery in patients with mobile vegetation length larger than 10 mm and embolic complication despite antibiotic therapy appropriate. It is accepted that large movable vegetation greater than 10 mm on the anterior leaflet of the mitral valve is associated with a higher risk of embolism. The mobility and length of vegetation, history of embolism, type of organism, size, location, and length of antibiotic treatment can affect the related risk of another embolism event [29, 30].

The studies and recent European guidelines on the prevention, diagnosis, and treatment of infective endocarditis showed that vegetation length >10 mm is the main risk factor of embolic events and mortality [1, 2, 3, 4]. In our study, we indicated that vegetation length ≥10 mm is associated with increased 1-year and 5-year mortality and identified vegetation length ≥10 mm to be associated with all-time mortality by using multivariate analysis of Cox regression. Follow-up data showed that almost of the deaths are in the first 1–2 years, which means all patients discharged must be followed up closely, particularly in the first 1–2 years.

It is very important for infective endocarditis to be timely diagnosed and treated. We advocate timely intervention of surgery, because infective endocarditis is progressive and life-threatening. There is always a dilemma when to conduct surgery: should we conduct surgery early to lessen the risk of embolism and gradual worsening of heart function, or should we conduct surgery following valid infection control to lessen the risk of surgery and complications? Timely surgical interventions during the acute phase of infective endocarditis, including shock, uncontrolled sepsis, and multi-organ failure, have raised concerns about high surgical mortality and risk of worsening. Delaying operations to complete a course of antibiotic treatment may increase embolism risk and result in extensive injury of cardiac tissue, leading to more challenging operation, progressive cardiogenic shock, and multi-organ failure, increasing mortality in the end. It is more aggressive and operation should be performed early on for patients at imminent embolism risk to achieve better early and late outcomes.

In most studies of left infective endocarditis, echocardiography is used to classify vegetation size into small (<5 mm), medium (5–9 mm), or large (≥10 mm). Vegetation size ≥10 mm is a predictive factor for increased embolic events and mortality. For larger vegetation—usually due to the inability of antibiotics to reduce the size of the vegetation during 4–8 weeks of treatment—as well as complications including formation of perivalvular abscess, valve injury, and persistent fever, intervention of surgery is required. Strong evidence indicates that vegetation length ≥10 mm is an indication for operating, particularly for left-sided infective endocarditis [4, 5, 18, 19, 20, 21].

Emergency surgery within 48 hours is rational for patients with mobile large vegetation at imminent risk of embolism [29, 30]. Early surgery is defined as a surgery that is performed independently of completing a complete course of antibiotics during the initial hospitalization. The surgery for preventing embolism is mainly related to the early stage, in the first few days after starting antibacterial treatment (urgent or urgent). According to the American Association for Thoracic Surgery (AATS) guidelines, once the surgical indication is determined, patients should undergo surgery as soon as possible, at least within a few days [1, 2, 3, 4, 17, 28, 29, 30].

In our observational study, multivariate analysis identified preoperative LVEF and aortic insufficiency to be associated with vegetation length ≥10 mm, which needs particular attention. First of all, it must be emphasized that, although statistically significant, from a practical point of view the mean LVEF values of 59.9% and 61.7% in the patient group with vegetation lengths <10 mm and ≥10 mm, respectively, revealed a difference of less than 3%. Given that both mean LVEF values are in a normal range, and that both the inter-observer and the intra-observer variability are usually higher than 3%, nobody could conclude that an LVEF of 62% might be associated with a vegetation of ≥10 mm, but a LVEF of 60% not. Secondly, given also that vegetations ≥10 mm appeared associated with higher mortality (which is absolutely comprehensible), the detection of an association between increasing normal preoperative LVEF values and the presence of vegetations ≥10 mm would misleadingly suggest an association of increasing normal LV pump function with higher mortality in patients with infective endocarditis (which is hardly imaginable). The explanation of the above mentioned contradictory findings is simply the fact that because of the higher prevalence of aortic regurgitation and mitral regurgitation (aortic regurgitation (AR) and mitral regurgitation (MR), respectively) in the majority of patients with vegetations ≥10 mm, the LVEF determined by volumetric calculation: LVEF(%) = (end diastolic volume, EDV – end systolic volume, ESV)/EDV × 100, where EDV and ESV are the end-systolic and end-diastolic volume, respectively, does not anymore reflect the fraction of chamber volume ejected into the aorta [16, 17]. Thus, in the presence of AR, a relevant part returns to the left ventricle (LV) during the diastole, whereas in the presence of MR a relevant part of the ejected blood is in fact delivered back to the left atrium. Therefore, in the presence of AR and/or MR the real LVEF(%) = EDV – (ESV + regurgitation volume)/EDV × 100 [18, 23]. This, in turn, indicates that the LVEF computed according to the biplane Simpson method will become unreliable and even misleading without including also the valve regurgitation into the ejection fraction (EF) calculation. This study is a good example of how relevant mitral regurgitation could be the misleading impact of valve insufficiency on the informative value of the LVEF in the clinical praxis by overestimating the LV pump function [17, 31].

However, in the study conducted by Li *et al*. [32], 201 consecutive patients (aged 64 ± 13 years, 74% male) were ultimately diagnosed with IE, and 14 patients had negative IE results. Vegetation size showed a high predictive ability for IE, with an optimal cut-off value of 11.5 mm. It should be clarified that in many cases, vegetation <10 mm during histological examination is not the true endocardial vegetation.

In our research, patients in the vegetation size <10 mm group did not die until at least 15 months after the start of follow-up, maybe indicating the prognostic value of vegetation size <10 mm in the long-term results.

### 4.3 Study Limitations 

One limitation of this study included its retrospective design. Due to the retrospective nature of the study and the role of our medical center as a tertiary referral hospital, there may be selection bias. The other limitation of the study was the lack of information regarding the impact of vegetation attachment and mobility.

## 5. Conclusions

Our study indicated that vegetation length greater than 10 mm is associated with long-term survival in patients treated surgically for infective endocarditis.

## Availability of Data and Materials

The datasets used and/or analysed during the current study are available from the corresponding author on reasonable request.

## Supplementary Material

Supplementary material associated with this article can be found, in the online version, at https://doi.org/10.31083/j.rcm2510354.
