# Supplementary material for: Vegetation Length is Associated with Long-term Survival in Patients Treated Surgically for Infective Endocarditis
Source: Rev Cardiovasc Med. 2024 Sep 30;25(10):354. doi: 10.31083/j.rcm2510354 (PMC11522749; doi:10.31083/j.rcm2510354)
Supplement: Supplementary file 1 [file 2153-8174-25-10-354-s1.docx]

**Supplementary Material**

Variables to be evaluated included age, gender (female / male), weight, time between symptoms and surgery, rheumatic heart disease, valvular heart disease, coronary heart disease, NYHA class, aortic insufficiency, mitral insufficiency, tricuspid insufficiency, left ventricular end diastolic dimension, left ventricular ejection fractions, serum creatinine, mean mechanical ventilation, ICU retention time, hospitalized time after surgery, postoperative chest drainage, packed red cells, fresh-frozen plasma, fluid balance on operation day, the first day following operation and the second day following operation, acute renal injury, multiorgan failure, long-term intubation, hepatic failure, respiratory failure, ventricular fibrillation, extracorporeal membrane oxygenation (ECMO) requirement, and death.
